# Protein Tyrosine Phosphatase 1B Inhibitors from the Stems of *Akebia quinata*

**DOI:** 10.3390/molecules21081091

**Published:** 2016-08-19

**Authors:** Jin-Pyo An, Thi Kim Quy Ha, Jinwoong Kim, Tae Oh Cho, Won Keun Oh

**Affiliations:** 1 Korea Bioactive Natural Material Bank, Research Institute of Pharmaceutical Sciences, College of Pharmacy, Seoul National University, Seoul 151-742, Korea; ntopjp77@gmail.com (J.-P.A.); htkquy@ctu.edu.vn (T.K.Q.H.); jwkim@snu.ac.kr (J.K.); 2 Marine Bio Research Center, Department of Life Science, Chosun University, Gwangju 501-759, Korea; tocho@chosun.ac.kr

**Keywords:** *Akebia quinata*, terpenes, protein tyrosine phosphatase 1B (PTP1B), breast cancer

## Abstract

PTP1B deficiency in mouse mammary tumor virus (MMTV)-NeuNT transgenic mice inhibited the onset of MMTV-NeuNT-evoked breast cancer, while its overexpression was observed in breast cancer. Thus, PTP1B inhibitors are considered chemopreventative agents for breast cancer. As part of our program to find PTP1B inhibitors, one new diterpene glycoside (**1**) and 13 known compounds (**2**–**14**) were isolated from the methanol extract of the stems of *Akebia quinata*. All isolates were identified based on extensive spectroscopic data analysis, including UV, IR, NMR and MS. Compounds **2**, **3**, **6**, **8** and **11** showed significant inhibitory effects on the PTP1B enzyme, with IC_50_ values ranging from 4.08 ± 1.09 to 21.80 ± 4.74 μM. PTP1B inhibitors also had concentration-dependent cytotoxic effects on breast cancer cell lines, such as MCF7, MDA-MB-231 and tamoxifen-resistant MCF7 (MCF7/TAMR) (IC_50_ values ranging from 0.84 ± 0.04 to 7.91 ± 0.39 μM). These results indicate that compounds **6** and **8** from *Akebia quinata* may be lead compounds acting as anti-breast cancer agents.

## 1. Introduction

Breast cancer is a threat to the health of women, ranking as the most common cancer in women globally, with more than 1,600,000 cases diagnosed in 2012 [1]. The American Association for Cancer Research reported that the number of breast cancer cases in the United States will increase by 50 percent in 2030 compared to 2011. Furthermore, the cost for care of breast cancer was the highest among all cancer types in the USA in 2010 at $16.5 billion [2]. Although several drugs, including adriamycin and taxotere, are used to treat breast cancer, many adverse effects, such as heart problems and reduction of white blood cell numbers, have been reported [1]. Thus, the discovery of anti-breast cancer agents is an urgent need.

Protein tyrosine phosphatase 1B (PTP1B), a negative insulin regulator, has been implicated in the signaling of breast cancer. PTP1B overexpression was observed in 72% of breast tumors compared to normal epithelium, and 38% of tumors showed maximal expression of PTP1B [3]. PTP1B siRNA-transfected cells significantly prevented insulin-like growth factor 2 (IGF-2)-induced MCF-cell migration, and the growth of MCF-7 cells was delayed when PTP1B was inhibited [4]. When PTP1B knockout mice were crossed with mouse mammary tumor virus (MMTV)-NeuNT mice, less than 40% of MMTV-NeuNT/PTP1^−/−^ mice developed breast tumors over a 3 year period, whereas MMTV-NeuNT/PTP1B^+/+^ mice had tumors with an average latency of 13 months [5].

PTP1B is also known as a novel target for acting against type 2 diabetes, not only against breast cancer. PTP1B is expressed ubiquitously in all insulin-responsive tissue and negatively regulates the insulin cascade. PTP1B knockout mice had enhanced insulin sensitivity and glucose homeostasis, preventing type 2 diabetes [6]. Insulin-induced phosphorylation at both the insulin receptor β-chain (IRβ) and insulin-receptor substrate-1 (IRS-1) was improved in free fatty acid (FFA)-insulin-induced hepatocyte model cells when PTP1B was reduced [7]. Therefore, finding a leading molecule for inhibiting PTP1B might help prevent the prevalence of not only breast cancer but also diabetes and obesity.

The stems of *Akebia quinata* (Hoult) Decne. were investigated for PTP1B inhibitory effects and cytotoxic activity. Previous phytochemical studies identified some of the constituents of *A. quinata* as triterpene glycosides, triterpenes, lignans, and phenylethanoid glycosides [8]. The biological activities of its dried stem have been reported to include anti-inflammatory, anti-obesity and anti-oxidant effects [9]. However, PTP1B inhibitory activity from this plant has not been reported. In this paper, we report one new diterpene glycoside (**1**) and 13 known compounds, including four diterpenes (**2**–**5**). Among them, compounds **2**, **3**, **6**, **8** and **11** exhibited significant PTP1B inhibitory activity. Furthermore, the inhibitory activities of the isolated compounds were examined against MCF-7, MDB-MB-231 and tamoxifen-resistant MCF7 (MCF7/TAMR) breast cancer cell lines.

## 2. Results

**Figure 1:**
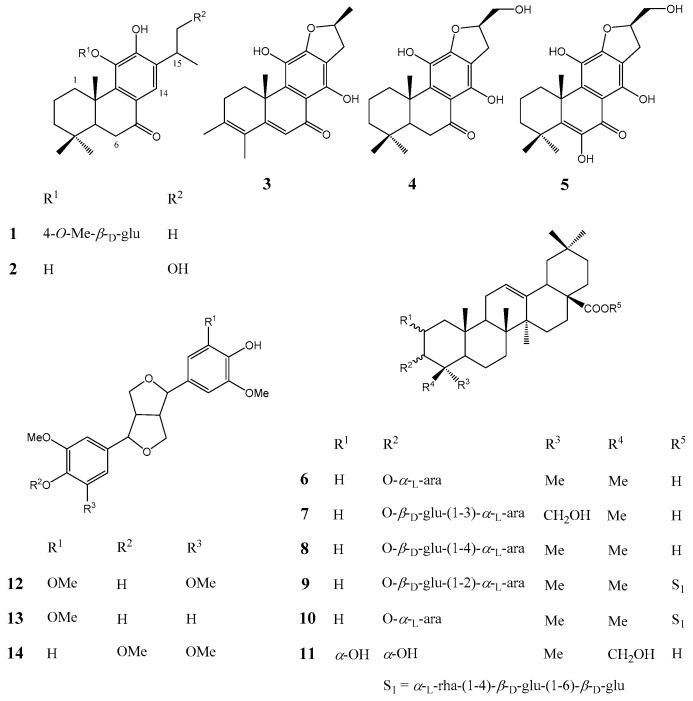
Chemical structures of compounds **1**–**14** isolated from the stems of *Akebia quinata*.

The EtOAc soluble fraction exhibited potential inhibitory activity of PTP1B at 20 μg/mL in a PTP enzyme assay. Activity-guided fractionations of EtOAc soluble fraction were performed by successive chromatographic procedures, including silica gel chromatography, Sephadex LH-20, RP-C_18_ and HPLC to afford one new diterpene glycoside (**1**) and 13 known compounds (**2**–**14**) (Figure 1).

**Figure 2:**
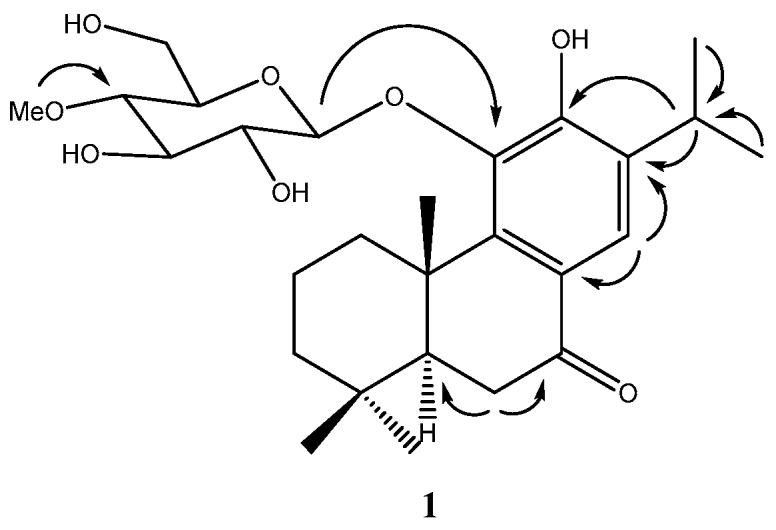
Key HMBC (from H to C) correlations for compound **1**.

Compound **1**, obtained as a yellowish gum, has a molecular formula of C_27_H_40_O_8_ as established by a quasimolecular ion in the HRESIMS at *m*/*z* 493.2782 [M + H]^+^ (calcd. for C_27_H_41_O_8_, 493.2796). The IR spectrum of compound **1** suggested the presence of hydroxyl (3287 cm^−1^), carbonyl (1666 cm^−1^) and phenyl (1593 cm^−1^) functionalities. The ^1^H-NMR spectrum showed signals for five methyl groups [δ_H_ 1.20 (3H, d, *J* = 6.9 Hz, H-16), 1.23 (3H, d, *J* = 6.9 Hz, H-17), 0.93 (3H, s, H-18), 1.02 (3H, s, H-19) and 1.57 (3H, s, H-20)], one aromatic single proton (δ_H_ 7.73, 1H, s, H-14) and one methine proton (δ_H_ 3.27, 1H, m, H-15) (Figure S1). The ^13^C-NMR spectrum revealed the presences of ketone carbon at δ_C_ 201.7 and one methoxy at δ_C_ 60.8 (Figure S2). The location of the ketone group at C-7 was confirmed by the HMBC correlations from H-14 (δ_H_ 7.73), H-6α (δ_H_ 2.61) and H-6β (δ_H_ 2.58) to C-7 (δ_C_ 201.7) (Figure S3). The presence of a hydroxyl group at C-12 was confirmed based on the HMBC correlations from H-14 (δ_H_ 7.73) to C-12 (δ_C_ 154.6) and from H-15 (δ_H_ 3.27) to C-12 (δ_C_ 154.6). One aromatic proton at C-14 was deduced by HMBC correlations from a single proton at δ_H_ 7.73 to C-7 (δ_C_ 201.7), C-9 (δ_C_ 148.5), C-11 (δ_C_ 143.8) and C-12 (δ_C_ 154.6) (Figure 2 and Figures S4–S7). The ^1^H- and ^13^C-NMR data suggested that compound **1** resembled with those of sugiol structure except for the presence of one additional sugar at the C-11 position. The β-glucopyranosyl unit at C-11 of compound **1** was confirmed by the HMBC correlation from the anomeric proton (δ_H_ 4.54, 1H, d, *J* = 7.7 Hz) to C-11 (δ_C_ 143.8). The presence of one methyl group at C-4′ (δ_C_ 80.3) of glucopyranosyl was also suggested by HMBC correlations from the methoxy (δ_H_ 3.58, 3H) to C-4′ (δ_C_ 80.3) (Figure 2). Thus, compound **1** was established as 11-*O*-(4-*O*-methyl-β-d-glucopyranosyl)-12-hydroxyabieta-8,11,13-triene-7-one (Table 1).

By comparison of the spectroscopic and optical rotation values with the literature data, the 13 known compounds were identified as cyrtophyllones B (**2**) [10], uncinatone (**3**) [10], villosin B (**4**) [11], villosin C (**5**) [11], 3-*O*-α-l-arabinopyranosyl olean-12-en-28-oic acid (**6**) [12], 3-*O*-[β-d-glucopyranosyl(1-3)-α-l-arabinopyranosyl)] hederagenin (**7**) [13], 3-*O*-[β-d-glucopyranosyl(1-4)-α-l-arabinopyranosyl)]olean-12-en-28-oic acid (**8**) [14], ciwujianoside A1 (**9**) [15], ciwujianoside C3 (**10**) [16], 2α,3α,23-trihydroxyoleane-12-en-28-oic acid (**11**) [17], (−)-syringaresinol (**12**) [18], medioresinol (**13**) [19], (2*R*,6*R*)-2-(4-hydroxy-3-methoxyphenyl)-6-(3,4,5-trimethoxypheny-l)-3,7-dioxabicyclo[3,3,0]octane (**14**) [20].

**Figure 3:**
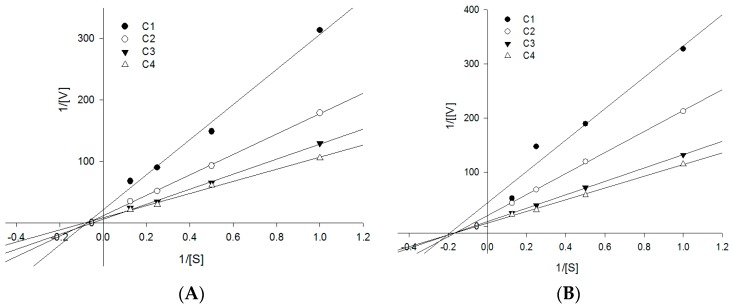
Graphical determination of the type of inhibition for compounds **3** and **6**. Lineweaver-Burk plots for the inhibition of compounds **3** (**A**) and **6** (**B**) on the PTP1B enzyme using *p*NPP assay. The conditions were as follows: 4 mM substrate, 0.05–0.1 μg/mL of PTP1B enzyme, 50 mM Tris buffer (pH 7.5), at room temperature. In the presence of different concentrations of compounds for lines from bottom to top: (**A**) for compound **3**, 0, 1.0, 2.0, 5.0, and 10.0 μM; (**B**) for compound **6**, 0, 1.0, 2.0, 5.0, and 10.0 μM. The data were evaluated in three replicates at each substrate concentration.

All isolated compounds were analyzed with respect to their inhibitory effects on PTP1B enzyme activity. Compounds **2**, **3**, **6**, **8** and **11** significantly inhibited PTP1B activity, with IC_50_ values ranging from 4.08 ± 1.09 to 21.80 ± 4.74 μM (Table 2). Compound **6** had an especially strong inhibitory effect on PTP1B enzyme, with an IC_50_ value of 4.08 ± 1.09 μM, which was similar to that of the positive control (ursolic acid; IC_50_ value: 4.25 ± 0.62 μM). As compounds **3** and **6** were potential PTP1B inhibitors, their inhibition mode was determined using a double reciprocal Lineweaver-Burk plot. Compounds **3** and **6** were found noncompetitive inhibitors because increasing the substrate concentrations resulted in a family of lines that intersect at a non-zero point on the negative *x*-axis (Figure 3). Compounds **2**, **3**, **6**, **8** and **11** with PTP1B inhibition, were analyzed for their inhibitory effects against MCF7, MDA-MB-231 and MCF7/TAMR breast cancer cell lines. Four compounds **2**, **3**, **6** and **8**, had strong cytotoxic activities against breast cancer lines, with IC_50_ values ranging from 0.84 ± 0.17 to 13.42 ± 1.26 μM, compared with positive controls tamoxifen and 4-hydroxy tamoxifen (IC_50_ values ranging from 2.11 ± 0.39 to 25.42 ± 1.79 μM) (Table 2). Interestingly, while the effectiveness of the positive controls, tamoxifen and 4-OH tamoxifen, decreased by 1/3 to 1/6 in the MCF7/TAMR cell line, compounds **6** and **8** showed similar cytotoxic effects between MCF7 and MCF7/TAMR cancer cells. These results suggest that compounds **6** and **8** could also be considered good inhibitors against tamoxifen-resistant cancer cell lines.

**Figure 4:**
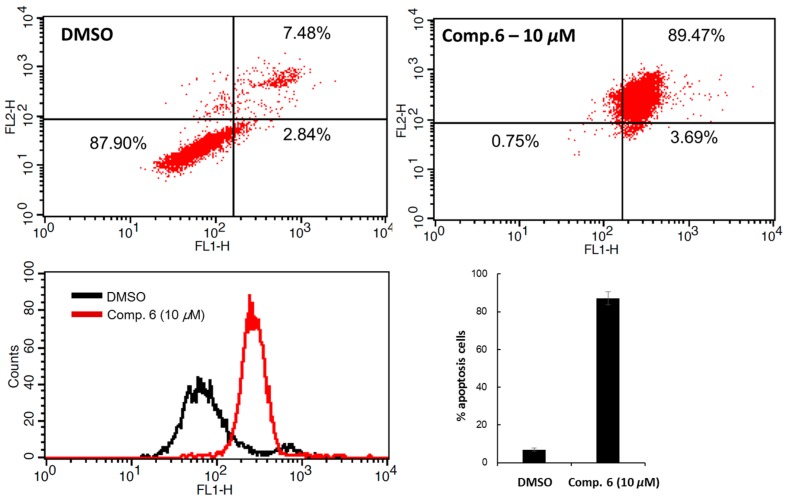
The inhibitory effect of compound **6** on MCF-7 cell apoptosis. The cells were treated with compound **6** (10 μM) and incubated for 12 h. Then, flow cytometric analysis was performed to detect cell apoptosis using the annexin conjugate ApopNexin™ FITC kit.

Apoptosis is the process of ordered and programmed cell death that occurs in both physiological and pathological conditions. As a chemopreventive strategy, apoptosis is considered one of the most promising treatments against breast cancer. Oblimersen, an apoptosis inducer, is presently in clinical trials with docetaxel, adriamycin and cyclophosphamide. Their preclinical and clinical potential, in combinations with many cancer medicines, against solid tumors is being used to discover therapeutic agents that effectively induce apoptosis [21]. The ability to induce apoptosis by compound **6** was determined by flow cytometry assay, using the ApopNexin™ FITC kit (Millipore, Billerica, MA, USA) to perform double-staining with Annexin FITC and propidium iodide (PI) in MCF-7 cells (with DMSO as a control). In the control group, 87.90% of cancer cells are viable, as shown in the lower left field (low Annexin V and PI staining). However, significant changes in the cancer cell profiles were observed when 10 μM of compound **6** was added. The percentage of apoptotic cancer cells after 10 μM treatment of compound **6** was increased to 89.47%, as shown in the higher right field (Figure 4). These results suggested that compound **6** increased cancer cell cytotoxicity mainly by inducing cell apoptosis.

From a structural perspective, triterpene compounds (**6**, **8** and **11**) with a COOH group at C-28 showed a significant enhancement of inhibitory activity against PTP1B enzyme. This result is consistent with previous studies that 28-COOH may be responsible for inhibiting the PTP1B enzyme [22]. In addition, compound **6** with one less glucopyranosyl moiety than that of compound **8**, had stronger activity than compound **8**. This increased activity of compound **6** might be due to having one less glucopyranosyl, which is attached to C-3′ of arabinopyranosyl. The presence of an alcohol group at C-23 may decrease the inhibitory effects on PTP1B. In agreement with a previous study, compound **8**, which does not possess a hydroxyl group at C-23, had stronger inhibitory activity than compound **7**. The same trend was also observed with diterpene compounds **3**, **4** and **5** (data not shown). In a previous study, kaurane-type diterpenes without a hydroxy group at C-16 showed enhanced activity on PTP1B enzyme [23]. Among abietane type diterpenes, three tanshinones from root of *Salvia miltiorrhiza* have been reported to have PTP1B inhibitory effects. On the basis of our research, abietane diterpenes (**2** and **3**) from *Akebia quinata* were also found to have inhibitory effects on PTP1B enzyme.

## 3. Experimental Section

### 3.1. General Information

NMR spectra were recorded on a JEOL 600 MHz spectrometer (JEOL, Tokyo, Japan) with TMS as the internal standard at the Seoul National University, Seoul, Korea. IR spectra (KBr) were recorded on a Nicolet 6700 FT-IR (Thermo Fisher Scientific, Waltham, MA, USA). HRESIMS data were obtained with Agilent 1260 Infinity Q-TOF mass spectrometer (Agilent Technologies, Santa Clara, CA, USA). Optical rotation values were determined on a JASCO P-2000 polarimeter (JASCO, Tokyo, Japan). Silica gel (63–200 μm particle size, Merck, Darmstadt, Germany), and RP-C_18_ (75 μm particle size, Nacalai Tesque, Kyoto, Japan) were used for column chromatography. TLC was carried out with silica gel 60 F_254_ and RP-C_18_ F_254_ plates. HPLC was performed on a Gilson System with an Optima Pak C_18_ column (10 μm particle size, 10 mm × 250 mm; RS Tech, Seoul, Korea) and a UV detector. Analytical grade solvents were purchased from Fisher scientific (Pittsburgh, PA, USA).

### 3.2. Extraction and Isolation

The stems of *A. quinata* were collected in September in 2014 at Gangwon province, Korea. The dried stem (10 kg) of *A. quinata* were extracted with 90% MeOH at room temperature for 1 week. The combined extract was concentrated under reduced pressure to yield a dried sample (400 g). This crude extract was then suspended in H_2_O (4 L) and partitioned successively with *n*-hexane (3 × 4 L), EtOAc (3 × 4 L), and *n*-BuOH (3 × 4 L). The partial EtOAc fraction (47 g) was chromatographed over a silica gel column (5 × 30 cm; 63–200 μm particle size) and eluted with gradient mixtures of *n*-hexane/acetone (10:1 → 1:10, each 2 L) to afford six sub-fractions (Fr.1 to Fr.6). Fr.6 (4.3 g) was applied onto an RP-18 column (4.5 × 50 cm; 75 μm particle size) using a MeOH/H_2_O system (1:1.5 to 1:0) to yield eleven sub-fractions and compound **6** (21 mg). Fr.6-9 (112 mg) was purified by HPLC (mobile phase 33% MeCN in H_2_O containing 0.1% formic acid; flow rate, 2 mL/min; UV detection at 205 and 254 nm) using an isocratic solvent system to give compound **7** (5.6 mg; *t*_R_ = 30.0 min). Fr.6-10 (150 mg) was further purified using HPLC (mobile phase MeCN/H_2_O containing 0.1% formic acid; 0–8 min: 10%–35% MeCN, 8–12 min: 35%–39% MeCN, 12–24 min: 39% MeCN, 25–34 min: 100% MeCN, 35–39 min: 10% MeCN; flow rate, 2 mL/min; UV detection at 205 and 254 nm) to afford compound **8** (10 mg; *t*_R_ = 23.2 min). Fr.6-11 (185 mg) was also purified by HPLC (mobile phase 33% MeCN in H_2_O containing 0.1% formic acid; 0–10 min: 60% MeOH, 10–15 min: 60%–75% MeOH, 15–45 min: 75%–81% MeOH, 45–55 min: 100% MeOH, 55–57 min: 60% MeOH; flow rate, 2 mL/min; UV detection at 205 and 254 nm) to yield compound **5** (5.0 mg; *t*_R_ = 38.3 min). Fr.5 (5 g) was chromatographed on an RP-18 column (4.5 × 55 cm; 75 μm particle size) and was eluted with a gradient MeOH/H_2_O system (1:1 to 1:0) to afford 5 sub-fractions. Further purification of subfraction Fr.5-2 (102 mg) by HPLC, using an isocratic solvent system of 20% MeCN in H_2_O containing 0.1% formic acid resulted in isolation of compound **2** (3.3 mg; *t*_R_ = 20.5 min). Fr.4 (8 g) was subjected to an RP-18 column (4.5 × 40 cm; 75 μm particle size) using MeOH/H_2_O system (1:1 to 1:0) to give 10 subfractions. Fr.4-10 (90 mg) was further purified by HPLC with an isocratic solvent system of 89% MeOH in H_2_O containing 0.1% formic acid to give compound **4** (13 mg; *t*_R_ = 30.7 min). Fr.3 (10 g) was fractionated into twelve subfractions (Fr.3-1 to Fr.3-12) and yielded compound **3** (32 mg) by RP-18 column (4.5 × 50 cm; 75 μm particle size) eluted with MeOH/ H_2_O (1:1 to 1:0). Fr.3-3 (32 mg) was purified by HPLC (mobile phase MeOH/H_2_O containing 0.1% formic acid; 0–7 min: 20% MeOH, 7–43 min: 28%–30% MeOH, 43–46 min: 30%–34% MeOH, 46–55 min: 34% MeOH, 55–64 min: 100% MeOH, 64–67 min: 20% MeOH; flow rate, 2 mL/min; UV detection at 205 and 254 nm) to give compounds **13** (3.0 mg; *t*_R_ = 45.1 min) and **14** (2.0 mg; *t*_R_ = 50.1 min). Fr.3-11 (180 mg) was purified by HPLC, using an isocratic solvent system of 53% MeCN in H_2_O containing 0.1% formic acid to give compounds **1** (3.8 mg; *t*_R_ = 22.1 min), **9** (2.5 mg; *t*_R_ = 33.7 min), **10** (7.8 mg; *t*_R_ = 52.5 min) and **11** (5.6 mg; *t*_R_ = 28.8 min). Fr 3–12 (105 mg) was purified by HPLC using an isocratic solvent system of 52% MeCN in H_2_O containing 0.1% formic acid to give compound **12** (4.5 mg; *t*_R_ = 39.7 min).

Compound **1**: Yellowish gum, ${\left[\alpha \right]}_{D}^{20}$ + 26.7 (*c* 0.1, MeOH) UV (MeOH) λ_max_ (log ε): 212 (2.72), 232 (2.36), 288 (2.72) nm; IR (KBr) νmax 3287, 2958, 2928, 2868, 1666, 1593, 1315, 1071, 1022 cm^−1^; HRESIMS positive-charged *m*/*z* 493.2782 [M + H]^+^ (calcd. for C_27_H_41_O_8_, 493.2796) (Figure S8). ^1^H-NMR (methanol-*d*_4_, 600 MHz) and ^13^C-NMR (methanol-*d*_4_, 600 MHz) see Table 1.

Compound **2**: Colorless needles, UV (MeOH), λ_max_ 212, 227, 290 nm; ^1^H-NMR (chloroform-*d*, 600 MHz) δ: 7.45 (1H, s, H-14), 4.00 (1H, m, H-16a), 3.76 (1H, m, H-16b), 3.26 (1H, d, 13.5 Hz, H-1a), 1.45 (1H, m, H-1b), 3.14 (1H, m, H-15), 1.35 (3H, s, H-20), 1.33 (3H, d, 6.0 Hz, H-17), 0.93 (3H, s, H-19), 0.90 (3H, s, H-18); ^13^C-NMR (chloroform-*d*, 600 MHz) δ: 37.9 (C-1), 19.0 (C-2), 41.3 (C-3), 33.5 (C-4), 50.9 (C-5), 35.6 (C-6), 199.4 (C-7), 125.3 (C-8), 138.6 (C-9), 40.1 (C-10), 143.3 (C-11), 147.4 (C-12), 128.5 (C-13), 119.1 (C-14), 36.2 (C-15), 69.5 (C-16), 15.2 (C-17), 33.2 (C-18), 21.5 (C-19), 18.0 (C-20).

Compound **3**: Yellow power, UV (MeOH), λ_max_ 225, 283, 304 nm; ^1^H-NMR (methanol-*d*_4_, 600 MHz) δ: 6.16 (1H, s, H-6), 5.13 (1H, ddq, 7.9, 6.9, 6.4 Hz, H-16), 3.43 (1H, dd, 13.2, 5.3 Hz, H-15a), 3.35 (1H, m, H-1a), 2.82 (1H, dd, 15.2, 7.3 Hz, H-15b), 1.93 (3H, s, H-18), 1.90 (3H, s, H-19), 1.51 (3H, s, H-20), 1.50 (3H, d, 6.4 Hz, H-17); ^13^C-NMR (methanol-*d*_4_, 600 MHz) δ: 30.3 (C-1), 31.2 (C-2), 142.4 (C-3), 126.3 (C-4), 168.0 (C-5), 118.8 (C-6), 191.6 (C-7), 109.9 (C-8), 138.5 (C-9), 41.2 (C-10), 133.5 (C-11), 154.7 (C-12), 112.6 (C-13), 156.8 (C-14), 34.9 (C-15), 83.6 (C-16), 22.3 (C-17), 20.6 (C-18), 15.1 (C-19), 22.2 (C-20).

Compound **4**: Yellow power, UV (MeOH), λ_max_ 225, 296 nm; ^1^H-NMR (methanol-*d*_4_, 600 MHz) δ: 5.03 (1H, m, H-16), 3.79 (1H, dd, 12.9, 4.3 Hz, H-17a), 3.69 (1H, dd, 12.1, 6.0 Hz, H-17b), 3.48 (1H, d, 13.4 Hz, H-1a), 3.20 (1H, d, 15.4, 9.5 Hz, H-15a), 2.97 (1H, dd, 15.4, 7.0 Hz, H-15b), 1.34 (1H, m, H-1b), 1.38 (3H, s, H-20), 0.99 (3H, s, 19), 0.96 (3H, s, H-18); ^13^C-NMR (methanol-*d*_4_, 600 MHz) δ: 37.6 (C-1), 20.1 (C-2), 42.4 (C-3), 34.4 (C-4), 51.7 (C-5), 36.3 (C-6), 206.3 (C-7), 111.4 (C-8), 142.3 (C-9), 42.1 (C-10), 133.4 (C-11), 158.7 (C-12), 112.0 (C-13), 156.4 (C-14), 29.3 (C-15), 87.3 (C-16), 64.9 (C-17), 33.7 (C-18), 22.0 (C-19), 18.0 (C-20).

Compound **5**: Yellow power, UV (MeOH), λ_max_ 225, 267 nm; ^1^H-NMR (chloroform-*d*, 600 MHz) δ: 5.13 (1H, m, H-16), 3.93 (1H, dd, 12.2, 2.6 Hz, H-17a), 3.83 (1H, dd, 12.1, 6.7 Hz, H-17b), 3.34 (1H, dd, 15.4, 9.5 Hz, H-15a), 3.05 (1H, dd, 15.3, 7.3 Hz, H-15b), 3.06 (1H, m, H-1a), 1.13 (1H, m, H-1b), 1.65 (3H, s, H-20), 1.44 (3H, s, H-19), 1.43 (3H, s, H-18); ^13^C-NMR (chloroform-*d*, 600 MHz) δ: 28.9 (C-1), 17.8 (C-2), 42.0 (C-3), 36.4 (C-4), 144.5 (C-5), 141.6 (C-6), 183.0 (C-7), 107.2 (C-8), 140.2 (C-9), 36.7 (C-10), 130.9 (C-11), 153.3 (C-12), 110.5 (C-13), 154.4 (C-14), 30.0 (C-15), 86.5 (C-16), 64.9 (C-17), 28.1 (C-18), 27.2 (C-19), 27.5 (C-20).

Compound **6**: White powder, UV (MeOH), λ_max_ 205 nm; ^1^H-NMR (pyridine-*d*_5_, 300 MHz) δ: 5.48 (1H, t-like, H-12), 4.78 (1H, d, 7.1 Hz, Ara-H-1), 1.29 (3H, s, H-27), 1.27 (3H, s, H-23), 1.00 (6H, s, H3-24,30), 0.99 (6H, s, H3-26,29), 0.85 (3H, s, H-25); ^13^C-NMR (pyridine-*d*_5_, 300 MHz) δ: 38.8 (C-1), 26.6 (C-2), 88.4 (C-3), 39.6 (C-4), 55.9 (C-5), 18.5 (C-6), 33.2 (C-7), 39.7 (C-8), 48.0 (C-9), 37.0 (C-10), 23.7 (C-11), 122.6 (C-12), 144.8 (C-13), 42.2 (C-14), 28.3 (C-15), 23.7 (C-16), 46.7 (C-17), 41.9 (C-18), 46.5 (C-19), 30.9 (C-20), 34.2 (C-21), 33.3 (C-22), 28.2 (C-23), 16.9 (C-24), 15.5 (C-25), 17.4 (C-26), 26.2 (C-27), 180.2 (C-28), 33.2 (C-29), 23.8 (C-30), 107.5 (Ara-1), 72.9 (Ara-2), 74.6 (Ara-3), 69.5 (Ara-4), 66.8 (Ara-5).

Compound **7**: White power, UV (MeOH), λ_max_ 205 nm; ^1^H-NMR (pyridine-*d*_5_, 600 MHz) δ: 5.48 (1H, t-like, H-12), 5.35 (1H, d, 7.6 Hz, Glc-H-1), 5.00 (1H, d, 7.4 Hz, Ara-H-1), 1.26 (3H, s, H-27), 1.02 (3H, s, H-25), 0.99 (3H, s, H-30), 0.93 (6H, s, H3-26,29), 0.92 (3H, s, H-24); ^13^C-NMR (pyridine-*d*_5_, 600 MHz) δ: 39.1 (C-1), 26.6 (C-2), 82.0 (C-3), 43.9 (C-4), 47.8 (C-5), 18.4 (C-6), 33.2 (C-7), 40.1 (C-8), 48.5 (C-9), 37.2 (C-10), 24.2 (C-11), 122.9 (C-12), 145.1 (C-13), 42.5 (C-14), 28.7 (C-15), 24.0 (C-16), 47.0 (C-17), 42.3 (C-18), 46.7 (C-19), 31.3 (C-20), 34.5 (C-21), 33.6 (C-22), 64.4 (C-23), 14.0 (C-24), 16.4 (C-25), 17.8 (C-26), 26.5 (C-27), 180.6 (C-28), 33.5 (C-29), 24.1 (C-30), 106.8 (Ara-1), 72.4 (Ara-2), 84.6 (Ara-3), 69.7 (Ara-4), 67.8 (Ara-5), 107.0 (Glc-1), 76.1 (Glc-2), 78.8 (Glc-3), 71.9 (Glc-4), 79.1 (Glc-5), 63.0 (Glc-6)

Compound **8**: White powder, UV (MeOH), λ_max_ 205 nm; ^1^H-NMR (methanol-*d*_4_, 400 MHz) δ: 5.24 (1H, t-like, H-12), 4.59 (1H, d, 7.8 Hz, Glc-H-1), 4.50 (1H, d, 6.0 Hz, Ara-H-1), 2.86 (1H, dd, 13.5, 3.8 Hz, H-18), 1.16 (3H, s, H-27), 1.06 (3H, s, H-23), 0.96 (3H, s, H-24), 0.94 (3H, s, H-30), 0.91 (3H, s, H-26), 0.85 (3H, s, H-29), 0.82 (3H, s, H-25); ^13^C-NMR (methanol-*d*_4_, 400 MHz) δ: 40.4 (C-1), 27.0 (C-2), 91.2 (C-3), 40.4 (C-4), 56.9 (C-5), 19.4 (C-6), 33.8 (C-7), 40.6 (C-8), 47.6 (C-9), 37.9 (C-10), 24.0 (C-11), 123.6 (C-12), 145.2 (C-13), 42.9 (C-14), 28.9 (C-15), 23.9 (C-16), 47.3 (C-17), 42.7 (C-18), 47.3 (C-19), 31.6 (C-20), 34.9 (C-21), 34.0 (C-22), 28.6 (C-23), 16.9 (C-24), 15.9 (C-25), 17.7 (C-26), 26.4, (C-27), 181.8 (C-28), 33.6 (C-29), 24.5 (C-30), 104.8 (Ara-1), 68.9 (Ara-2), 73.7 (Ara-3), 79.1 (Ara-4), 65.2 (Ara-5), 105.4 (Glc-1), 75.9 (Glc-2), 78.2 (Glc-3), 71.7 (Glc-4), 77.9 (Glc-5), 62.9 (Glc-6).

Compound **9**: White powder, UV (MeOH), λ_max_ 205 nm; ^1^H-NMR (methanol-*d*_4_, 600 MHz) δ: 5.34 (1H, d, 8.0 Hz, Glc′-H-1), 5.26 (1H, t-like, H-12), 4.89 (1H, s, Rha-H-1), 4.59 (1H, d, 7.7 Hz, Glc′′-H-1), 4.52 (1H, d, 6.0 Hz, Ara-H-1), 4.41 (1H, d, 7.9 Hz, Glc-H-1), 1.27 (3H, d, 6.2 Hz, Rha-H-6), 1.15 (3H, s, H-27), 1.05 (3H, s, H-23), 0.96 (3H, s, H-30), 0.95 (3H, s, H-24), 0.91 (3H, s, H-26), 0.85 (3H, s, H-29), 0.79 (3H, s, H-25); ^13^C-NMR (methanol-*d*_4_, 600 MHz) δ: 39.8 (C-1), 27.0 (C-2), 91.2 (C-3), 40.4 (C-4), 56.9 (C-5), 19.4 (C-6), 33.9 (C-7), 40.7 (C-8), 49.0 (C-9), 37.9 (C-10), 24.1 (C-11), 123.8 (C-12), 144.9 (C-13), 42.9 (C-14), 28.9 (C-15), 24.0 (C-16), 48.1 (C-17), 42.5 (C-18), 47.3 (C-19), 31.6 (C-20), 34.9 (C-21), 33.5 (C-22), 28.6 (C-23), 16.9 (C-24), 16.1 (C-25), 17.8 (C-26), 26.3 (C-27), 178.1 (C-28), 33.3 (C-29), 24.6 (C-30), 105.4 (Ara-1), 79.6 (Ara-2), 72.2 (Ara-3), 68.9 (Ara-4), 104.2 (Glc-1), 75.9 (Glc-2), 78.1 (Glc-3), 71.7 (Glc-4), 78.0 (Glc-5), 62.9 (Glc-6), 95.8 (Glc′-1), 73.8 (Glc′-2), 78.2 (Glc′-3), 70.9 (Glc′-4), 78.2 (Glc′-5), 69.4 (Glc′-6), 104.8 (Glc′′-1), 75.3 (Glc′′-2), 76.8 (Glc′′-3), 79.0 (Glc′′-4), 76.7 (Glc′′-5), 61.9 (Glc′′-6), 103.0 (Rha-1), 73.7 (Rha-2), 72.4 (Rha-3), 73.9 (Rha-4), 70.7 (Rha-5), 17.9 (Rha-6).

Compound **10**: White powder, UV (MeOH), λ_max_ 205 nm; ^1^H-NMR (pyridine-*d*_5_, 600 MHz) δ: 6.28 (1H, d, 8.4 Hz, Glc-H-1), 5.90 (1H, s, Rha-H-1), 5.42 (1H, s, H-12), 5.02 (1H, d, 7.8 Hz, Glc′-H-1), 4.79 (1H, d, 6.6 Hz, Ara-H-1), 1.72 (3H, d, 6.2 Hz, Rha-H-6), 1.25 (3H, s, H-27), 1.24 (3H, s, H-23), 1.15 (3H, s, H-30), 1.00 (3H, s, H-24), 0.90 (9H, s, H3-25,26,29); ^13^C-NMR (pyridine-*d*_5_, 600 MHz) δ: 39.1 (C-1), 27.0 (C-2), 89.0 (C-3), 39.9 (C-4), 56.2 (C-5), 18.9 (C-6), 33.5 (C-7), 40.0 (C-8), 48.4 (C-9), 37.4 (C-10), 24.2 (C-11), 123.2 (C-12), 144.5 (C-13), 42.5 (C-14), 28.6 (C-15), 24.0 (C-16), 47.4 (C-17), 42.0 (C-18), 46.5 (C-19), 31.1 (C-20), 34.3 (C-21), 32.9 (C-22), 28.6 (C-23), 17.3 (C-24), 15.9 (C-25), 17.9 (C-26), 26.4 (C-27), 176.9 (C-28), 33.4 (C-29), 24.0 (C-30), 108.0 (Ara-1), 74.2 (Ara-2), 75.1 (Ara-3), 70.0 (Ara-4), 67.3 (Ara-5), 96.0 (Glc-1), 74.4 (Glc-2), 79.1 (Glc-3), 71.2 (Glc-4), 78.4 (Glc-5), 69.5 (Glc-6), 105.3 (Glc′-1), 75.7 (Glc′-2), 76.9 (Glc′-3), 78.5 (Glc′-4), 77.6 (Glc′-5), 61.6 (Glc′-6), 103.1 (Rha-1), 72.9 (Rha-2), 73.2 (Rha-3), 73.3 (Rha-4), 70.7 (Rha-5), 18.9 (Rha-6).

Compound **11**: White powder, UV (MeOH), λ_max_ 205 nm; ^1^H-NMR (DMSO-*d*_6_, 300 MHz) δ: 5.16 (1H, t-like, H-12), 4.02 (1H, m, H-2), 3.92 (1H, m, H-3), 1.09 (3H, s, H-27), 0.93 (3H, s, H-25), 0.87 (6H, s, H3-26,30), 0.85 (3H, s, H-29), 0.67 (3H, s, H-24); ^13^C-NMR (DMSO-*d*_6_, 300 MHz) δ: 43.1 (C-1), 66.3 (C-2), 74.3 (C-3), 42.3 (C-4), 49.6 (C-5), 19.0 (C-6), 33.8 (C-7), 40.1 (C-8), 48.3 (C-9), 38.6 (C-10), 24.2 (C-11), 122.5 (C-12), 144.9 (C-13), 42.1 (C-14), 28.3 (C-15), 24.0 (C-16), 46.7 (C-17), 42.1 (C-18), 45.2 (C-19), 31.0 (C-20), 34.3 (C-21), 34.1 (C-22), 23.9 (C-23), 65.3 (C-24), 17.6 (C-25), 17.6 (C-26), 26.2 (C-27), 180.4 (C-28), 33.3 (C-29), 23.9 (C-30).

Compound **12**: Colorless needle, UV (MeOH), λ_max_ 237, 273 nm; ^1^H-NMR (methanol-*d*_4_, 300 MHz) δ: 6.60 (4H, s, H-2′,6′,2′′,6′′), 4.70 (2H, d, H-2,6), 4.20 (2H, dd, 8.7, 6.9 Hz, H-4a,8a), 3.80 (2H, dd, 9.1, 3.6 Hz, H-4b,8b), 3.83 (12H, s, OMe), 3.10 (2H, dd, 6.4, 4.6 Hz, H-1,5); ^13^C-NMR (methanol-*d*_4_, 300 MHz) δ: 54.3 (C-1), 86.0 (C-2), 71.8 (C-4), 54.3 (C-5), 86.0 (C-6), 71.8 (C-8), 132.0 (C-1′), 102.7 (C-2′), 147.1 (C-3′), 134.3 (C-4′), 147.1 (C-5′), 102.7 (C-6′), 132.0 (C-1′′), 102.7 (C-2′′), 147.1 (C-3′′), 134.3 (C-4′′), 147.1 (C-5′′), 102.7 (C-6′′).

Compound **13**: Colorless needle, UV (MeOH), λ_max_ 231, 280 nm; ^1^H-NMR (methanol-*d*_4_, 300 MHz) δ: 6.96 (1H, d, 1.5 Hz, H-6′′), 6.83 (1H, dd, 8.5, 1.5 Hz, H-2′′), 6.78 (1H, d, 8.5 Hz, H-3′′), 6.66 (2H, s, H-2′,6′), 4.72 (2H, d, 4.0 Hz, H-2,6), 4.27 (2H, m, H-4a,8a), 3.87 (3H, s, OMe-5′′), 3.85 (6H, s, OMe-3′,5′), 3.82 (2H, m, H-4b,8b), 3.16 (2H, m, H-1,5); ^13^C-NMR (methanol-*d*_4_, 300 MHz) δ: 55.2 (C-1), 87.5 (C-2), 72.5 (C-4), 55.4 (C-5), 87.4 (C-6), 72.6 (C-8), 133.0 (C-1′), 104.3 (C-2′), 149.2 (C-3′), 136.0 (C-4′), 149.2 (C-5′), 104.3 (C-6′), 133.6 (C-1′′), 120.0 (C-2′′), 116.0 (C-3′′), 147.2 (C-4′′), 149.0 (C-5′′), 111.0 (C-6′′), 56.6 (OMe-3′,5′), 56.2 (OMe-5′′).

Compound **14**: Colorless needle, UV (MeOH), λ_max_ 210, 280 nm; ^1^H-NMR (chloroform-*d*, 600 MHz) δ: 6.94 (1H, s, H-2′), 6.85 (1H, d, 8.0 Hz, H-5′), 6.73 (1H, d, 8.0, H-6′); ^13^C-NMR (chloroform-*d*, 600 MHz) δ: 54.4 (C-1), 85.8 (C-2), 72.0 (C-3), 54.4 (C-5), 87.1 (C-6), 72.6 (C-8), 131.3 (C-1′), 108.4 (C-2′), 146.6 (C-3′), 144.9 (C-4′), 114.2 (C-5′), 118.8 (C-6′), 137.6 (C-1′′), 102.8 (C-2′′), 153.5 (C-3′′), 137.6 (C-4′′), 153.5 (C-5′′), 102.8 (C-6′′). 60.6, 56.3, 56.3, 56.0 (OMe).

### 3.3. PTP1B Enzyme Assay

PTP1B (human, recombinant) was purchased from BIOMOL International LP (USA) and the enzyme activity was measured using *p*-nitrophenyl phosphate (*p*-NPP) (N1891, Sigma-Aldrich, St. Louis, MO, USA) as a substrate. In general, the assay was performed in 96-well plate containing 4 mM *p*-NPP and PTP1B (0.05–0.1 μg) in enzyme buffer (50 mM citrate (pH 6.0), 0.1 M NaCl, 1 mM dithiothreitol (DTT), and 1 mM EDTA) with or without test compounds. The assay was also carried out in the absence of PTP1B enzyme to exclude the effect of test compounds on the measurement absorbance. Following incubation at 37 °C for 30 min, the reaction was terminated with 10 M NaOH solution. The amount of produced *p*-nitrophenol was estimated by measuring the absorbance at 405 nm using an absorbance microplate reader (VersaMax™, Randor, PA, USA) [24]. For the enzyme kinetic determination, Lineweaver-Burk was used to determine the inhibition type of PTP1B. On the basis of Lineweaver-Burk double reciprocal plots, the PTP1B inhibition mode was obtained at various concentrations of p-NPP substrate (1, 2, 4, and 8 mM) without or with different test compound concentrations (1, 2, 5, and 10 μM). The 50% inhibition concentration (IC_50_) and inhibition type were evaluated using Sigma Plot Statistical Analysis software (11.0 software, SPCC Inc., Chicago, IL, USA).

### 3.4. Flow Cytometric Analysis of the Cell Apoptosis

An ApopNexin™ FITC kit (Millipore) was used for the flow cytometric analysis. After MCF-7 cells were seeded on 6-well plates and incubated for 24 h, the cells were treated with test compounds and incubated for 12 h. The detached cells were collected and resuspended to wash the cells twice with PBS. The cells were suspended in cold binding buffer (10 mM Hepes/NaOH pH 7.4, 140 mM NaCl, 2.5 mM CaCl_2_) at a concentration of 10^6^ cells/mL. Annexin conjugate ApopNexin™ FITC and propidium iodide (PI) were added to the cell suspension, which was incubated for 15 min in the dark. Then, the double-stained cells were immediately analyzed using a flow cytometer (BD bioscience, San Jose, CA, USA).

## 4. Conclusions

In this study, bioassay-guided fractionation of a methanol extract from *Akebia quinata* stems yielded one new diterpene glycoside (**1**) and 13 known compounds (**2**–**14**). Five compounds (**2**, **3**, **6**, **8** and **11**) exhibited potential inhibitory effects on PTP1B enzyme activity and cytotoxic effects on breast cancer cell lines. Using a flow-cytometry assay, compound **6** was further found to induce apoptosis in breast cancer cells. Therefore, these compounds may be developed as PTP1B inhibitors and anti-breast cancer agents.

## Figures and Tables

**Table 1:** ^1^H-NMR (methanol-*d*_4_, 600 MHz), ^13^C-NMR (methanol-*d*_4_, 150 MHz) data of compound **1** (δ in ppm, *J* in Hz).

No.	δ_H_ *J* (Hz)	δ_C_	HMBC	No.	δ_H_ *J* (Hz)	δ_C_	HMBC
1	3.47 (1H, d, *J* = 13.5 Hz)	38.3	C-2, C-3, C-5	15	3.27 (1H, m)	28.2	C-12, C-13, C-14, C-17
	1.40 (1H, m)						
2	1.81 (1H, dt, *J* = 3.7, 16.6 Hz)	20.1	C-3, C-5	16	1.20 (3H, d, *J* = 6.9 Hz)	22.8	C-15, C-17
	1.56 (1H, m)						
3	1.48 (1H, d, *J* = 15.8 Hz)	42.4	C-2, C-4, C-5	17	1.23 (3H, d, *J* = 6.9 Hz)	22.5	C-15,C-16
	1.29 (1H, dd, *J* = 4.6, 16.1 Hz)						
4		34.5		18	0.93 (3H, s)	33.5	C-3, C-4, C-5, C-19
5	1.76 (1H, dd, *J* = 3.9, 13.9 Hz)	51.7	C-3, C-4, C-6, C-7	19	1.02 (3H, s)	22.0	C-3, C-4, C-5, C-18
6	2.61 (1H, m)	36.3	C-5, C-7, C-8	20	1.57 (3H, s)	20.5	C-1, C-3, C-5, C-9
	2.58 (1H, m)						
7		201.7		1′	4.54 (1H, d, *J* = 7.7 Hz)	106.8	C-2′, C-11
8		124.8		2′	3.54 (1H, m)	75.9	C-1′, C-3′
9		148.5		3′	3.55 (1H, m)	78.1	C-2’, C-4’
10		40.4		4′	3.22 (1H, m)	80.3	C-3’, C-5’
11		143.8		5′	3.22 (1H, m)	77.9	C-4’, C-6’
12		154.6		6′	3.81 (1H, d, *J* = 11.3 Hz)	61.9	C-4’, C-5’
					3.71 (1H, d, *J* = 11.3 Hz)		
13		136.1		4′-OCH_3_	3.58 (3H, s)	60.8	C-4’
14	7.73 (1H, s)	123.7	C-7, C-9, C-11, C-12				

**Table 2:** Inhibitory effects of compounds **2**, **3**, **6**, **8** and **11** on PTP1B enzyme activity and their cytotoxic activities against breast cancer cell lines.

Compounds	PTP1B (IC_50_, μM) ^a^	Cytotoxic Activities (IC_50_, μM) ^a^		
		MCF7	MDA-MB-231	MCF7/TAMR
**2**	6.77 ± 1.28	7.91 ± 0.39	4.04 ± 0.80	13.42 ± 1.26
**3**	5.41 ± 0.68	6.02 ± 2.32	5.14 ± 1.55	7.73 ± 1.02
**6**	4.08 ± 1.09	1.11 ± 0.04	0.84 ± 0.17	1.39 ± 0.18
**8**	21.80 ± 4.74	1.29 ± 0.09	1.55 ± 0.21	1.78 ± 0.13
**11**	7.78 ± 1.43	>40	NT	NT
Ursolic acid ^b^	4.25 ± 0.62	NT	NT	NT
Tamoxifen ^b^	NT	8.01 ± 1.81	10.09 ± 1.51	25.42 ± 1.79
4-OH tamoxifen ^b^	NT	2.11 ± 0.39	3.10 ± 0.31	13.58 ± 1.25

NT: not tested, ^a^ Values are expressed as mean ± SD of three replicates, ^b^ Positive control.

## Supplementary Materials

Spectral data (^1^H-, ^13^C-NMR, HSQC and HMBC) of compound **1** are available free of charge on the Internet at http://www.mdpi.com/1420-3049/21/8/1091/s1.

## References

[1] Vasudevan D., Jayalakshmy P.S., Kumar S., Mathew S. (2015). Assessment of pathological response of breast carcinoma in modified radical mastectomy specimens after neoadjuvant chemotherapy. Int. J. Breast Cancer.

[2] Mariotto A.B., Yabroff K.R., Shao Y., Feuer E.J., Brown M.L. (2011). Projections of the cost of cancer care in the United States: 2010–2020. J. Natl. Cancer Inst..

[3] Wiener J.R., Kerns B.J.M., Harvey E.L., Conaway M.R., Iglehart J.D., Berchuck A., Bast R.C. (1994). Overexpression of the protein-tyrosine-phosphatase 1B in human breast-cancer-association with P185(C-Erbb-2) protein expression. J. Natl. Cancer Inst..

[4] Blanquart C., Karouri S.E., Issad T. (2010). Protein tyrosine phosphatase-1B and T-cell protein tyrosine phosphatase regulate IGF-2-induced MCF-7 cell migration. Biochem. Biophys. Res. Commun..

[5] Mohamed B.A., Benjamin G.N. (2007). Protein-tyrosine phosphatase 1B is required for HER2/*Neu*–induced breast cancer. Cancer Res..

[6] Gonzalez-Rodriguez A., Mas Gutierrez J.A., Sanz-Gonzalez S., Ros M., Burks D.J., Valverde A.M. (2010). Inhibition of PTP1B restores IRS1-mediated hepatic insulin signaling in IRS2-deficient mice. Diabetes.

[7] Ma Y.M., Tao R.Y., Liu Q., Li J., Tian J.Y., Zhang X.L., Xiao Z.Y., Ye F. (2011). PTP1B inhibitor improves both insulin resistance and lipid abnormalities in vivo and in vitro. Mol. Cell. Biochem..

[8] Mimaki Y., Doi S., Kuroda M., Yokosuka A. (2007). Triterpene glycosides from the stems of *Akebia quinata*. Chem. Pharm. Bull..

[9] Koo H.J., Sung Y.Y., Kim H.K. (2013). Inhibitory effects of *Akebia quinata* ethanol extract on TNF-α-mediated vascular inflammation in human aortic smooth muscle cells. Mol. Med. Rep..

[10] Tian X., Min Z., Xie N., Lei Y., Tian Z., Zheng Q., Xu R., Tanaka T., Iinuma M., Mizuno M. (1993). Abietane diterpenes from *Clerodendron cyrtophyllum*. Chem. Pharm. Bull..

[11] Ulubelen A., Topcu G., Olcal S. (1994). Rearranged abietane diterpenes from *Teucrium divaricatum* Subsp. Villosum. Phytochemistry.

[12] Morgan A.M.A., Kim J.H., Lee H.W., Lee S.H., Lim C., Jang H., Kim Y.H. (2015). Phytochemical constituents from the aerial part of *Ducrosia ismaelis* Asch. Nat. Prod. Sci..

[13] Martin P.K.C., Laurence V.N. (2005). Synthesis of l-arabinopyranose containing hederagenin saponins. Tetrahedron.

[14] Grishkovets V.I., Sobolev E.A., Shashkov A.S., Chirva V.Y. (2000). Triterpenoid glycosides of *Fatsia japonica*. II. Isolation and structure of glycosides from the leaves. Chem. Nat. Compd..

[15] Shao C.J., Kasai R., Xu J.D., Tanaka O. (1989). Saponins from leaves of *Acanthopanax senticosus* Harms., ciwujia. II.: Structures of ciwujianosides A_1_, A_2_, A_3_, A_4_ and D_3_. Chem. Pharm. Bull..

[16] Shao C.J., Kasai R., Xu J.D., Tanaka O. (1988). Saponins from leaves of *Acanthopanax senticosus* HARMS., ciwujia: Structures of ciwujianosides B, C_1_, C_2_, C_3_, C_4_, D_1_, D_2_ and E. Chem. Pharm. Bull..

[17] Ullah F., Hussain H., Hussain J., Bukhari I.A., Khan M.T., Choudhary M.I., Gilani A.H., Ahmad V.U. (2007). Tyrosinase inhibitory pentacyclic triterpenes and analgesic and spasmolytic activities of methanol extracts of *Rhododendron collettianum*. Phytother. Res..

[18] Chen B.N., Yang G.E., Li J.K., Du H.J., Li Q.S., Zhang Z.M. (2009). Cytotoxic constituents from *Viscum coloratum*. Chem. Nat. Compd..

[19] Tsukamoto H., Hisada S., Nishibe S. (1984). Lignans from bark of *Fraxinus mandshurica* var. *japonica* and *F. japonica*. Chem. Pharm. Bull..

[20] Miyazawa M., Kasahara H., Kameoka H. (1992). Phenolic lignans from flower buds of *Magnolia fargesii*. Phytochemistry.

[21] Wu J. (1996). Apoptosis and angiogenesis: Two promising tumor markers in breast cancer (review). Anticancer Res..

[22] Na M., Cui L., Min B.S., Bae K., Yoo J.K., Kim B.Y., Oh W.K., Ahn J.S. (2006). Protein tyrosine phosphatase 1B inhibitory activity of triterpenes isolated from *Astilbe koreana*. Bioorg. Med. Chem. Lett..

[23] Na M., Oh W.K., Kim Y.H., Cai X.F., Kim S.H., Kim B.Y., Ahn J.S. (2006). Inhibition of protein tyrosine phosphatase 1B by diterpenoids isolated from *Acanthopanax koreanum*. Bioorg. Med. Chem. Lett..

[24] Burke T.R., Ye B., Yan X., Wang S., Jia Z., Chen L., Zhang Z.Y., Barford D. (1996). Small molecule interactions with protein-tyrosine phosphatase PTP1B and their use in inhibitor design. Biochemistry.

