# Supplementary material for: Protein Tyrosine Phosphatase 1B Inhibitors from the Stems of Akebia quinata
Source: Molecules. 2016 Aug 19;21(8):1091. doi: 10.3390/molecules21081091 (PMC6273847; doi:10.3390/molecules21081091)
Supplement: Supplementary file 1 [file molecules-21-01091-s001.pdf]

Supplementary Materials: Protein Tyrosine Phosphatase 1B Inhibitors from the Stems of *Akebia quinata*

Jin-Pyo An, Thi Kim Quy Ha, Jinwoong Kim, Tae Oh Cho and Won Keun Oh

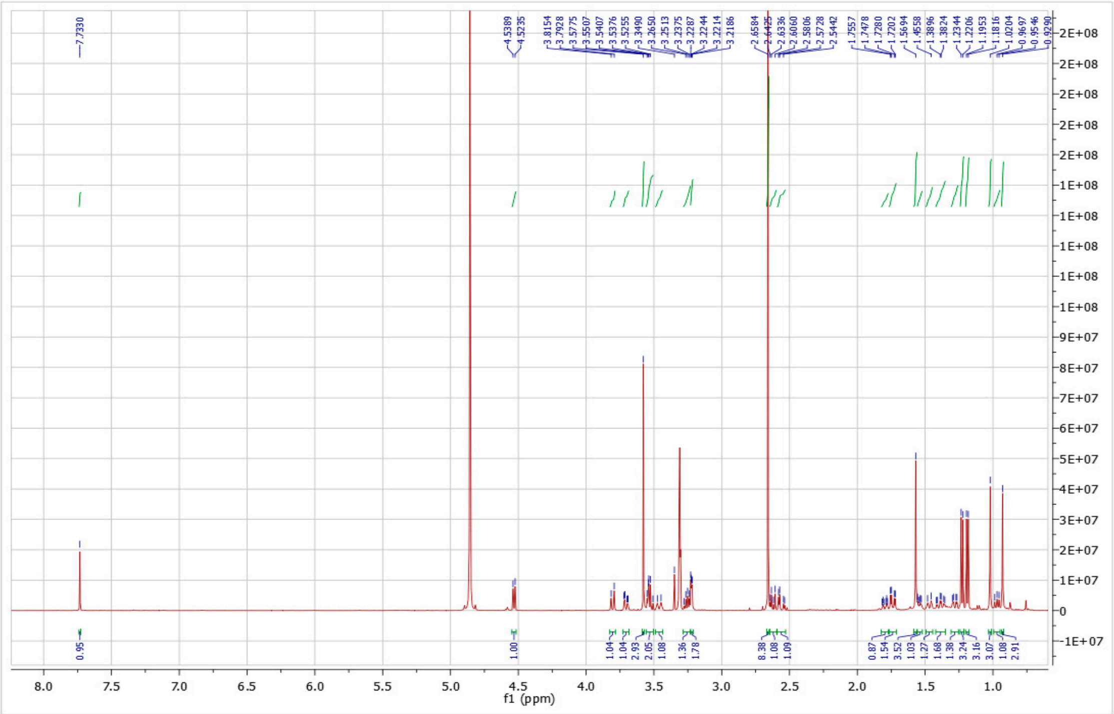

Figure S1. ^{1}H NMR spectrum of compound **1** in methanol-d_4 at 600 MHz.

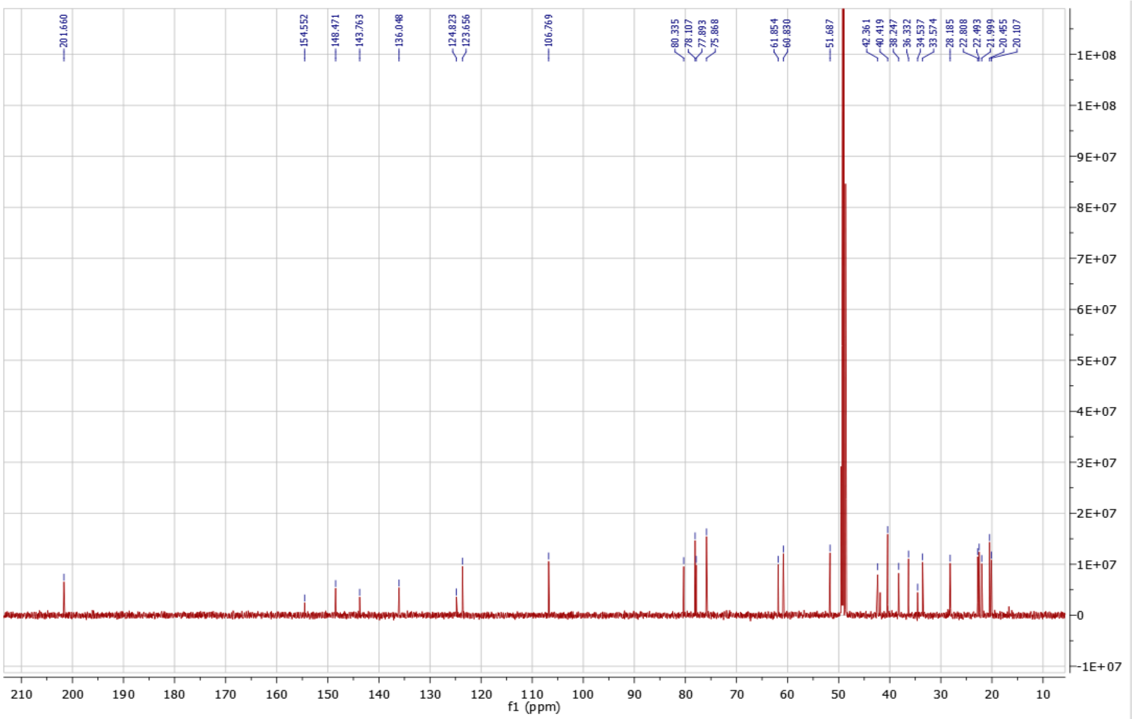

Figure S2. ^{13}C NMR spectrum of compound **1** in methanol-d_4 at 600 MHz.

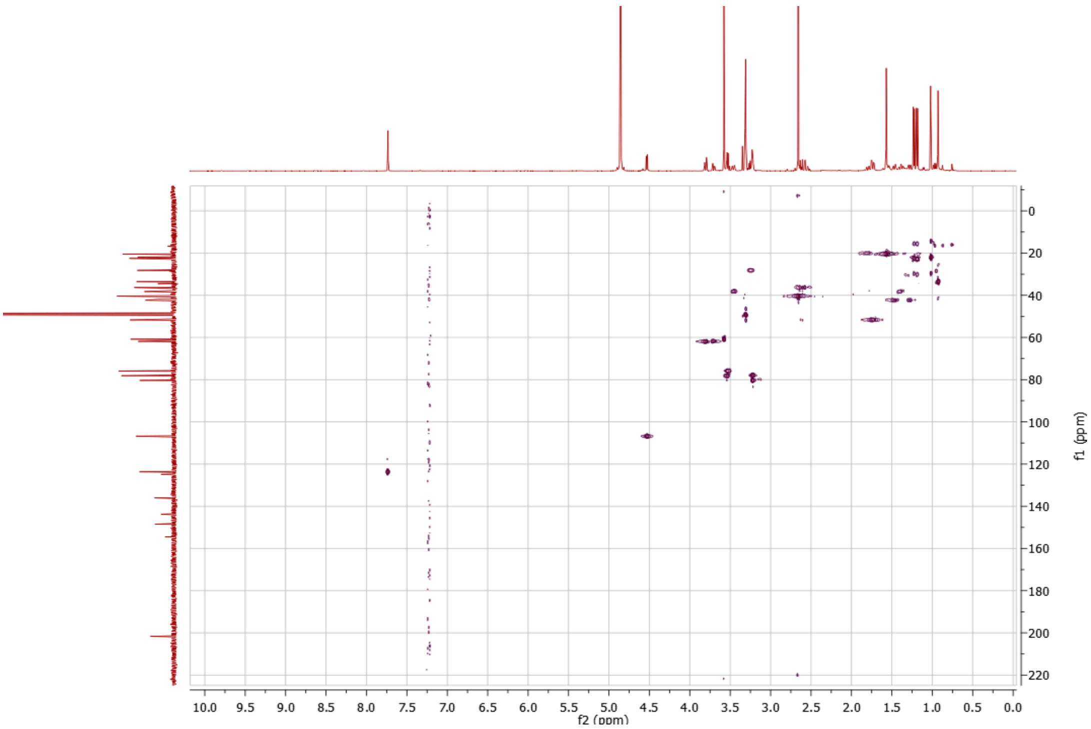

Figure S3. HSQC NMR spectrum of compound **1** in methanol-d_4 at 600 MHz.

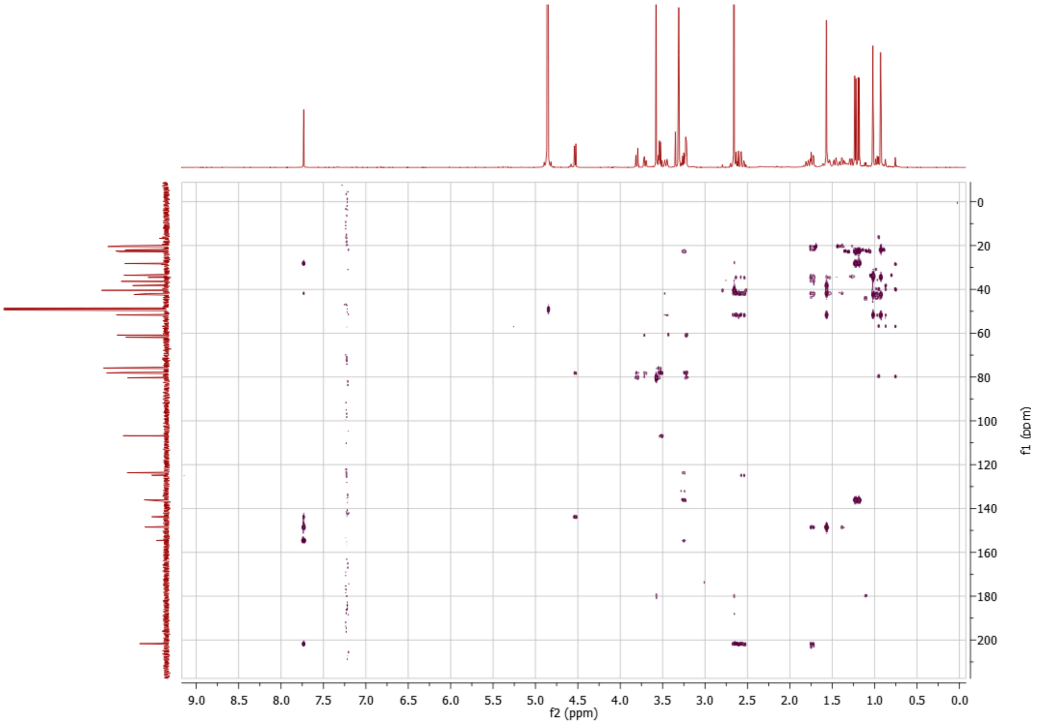

Figure S4. HMBC NMR spectrum of compound **1** in methanol-d_4 at 600 MHz.

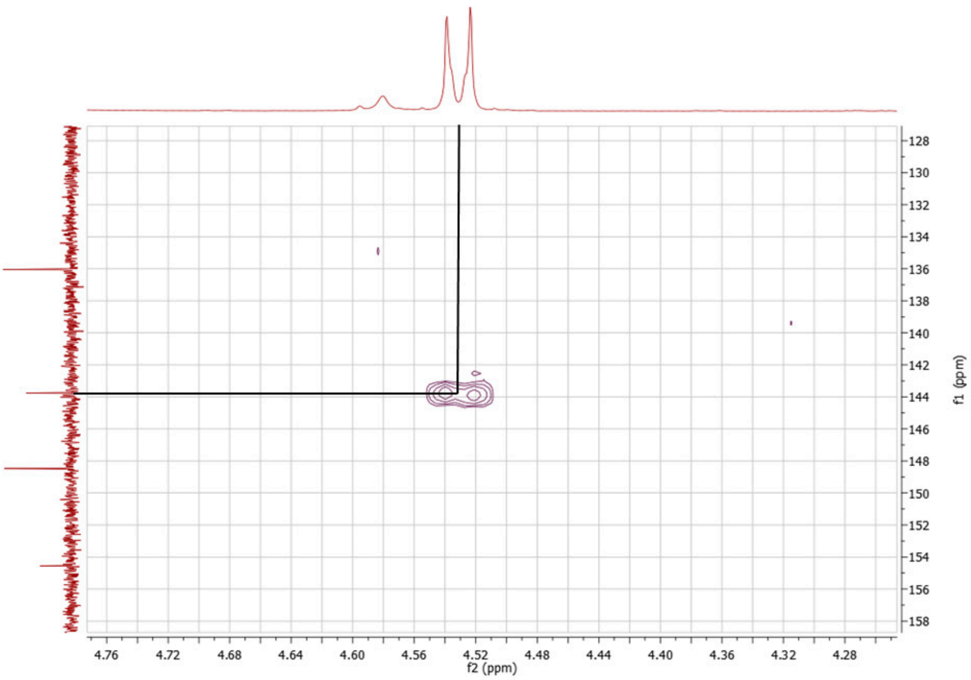

Figure S5. HMBC correlation from H-1′ to C-11 of compound **1**in methanol-*d*4 at 600 MHz.

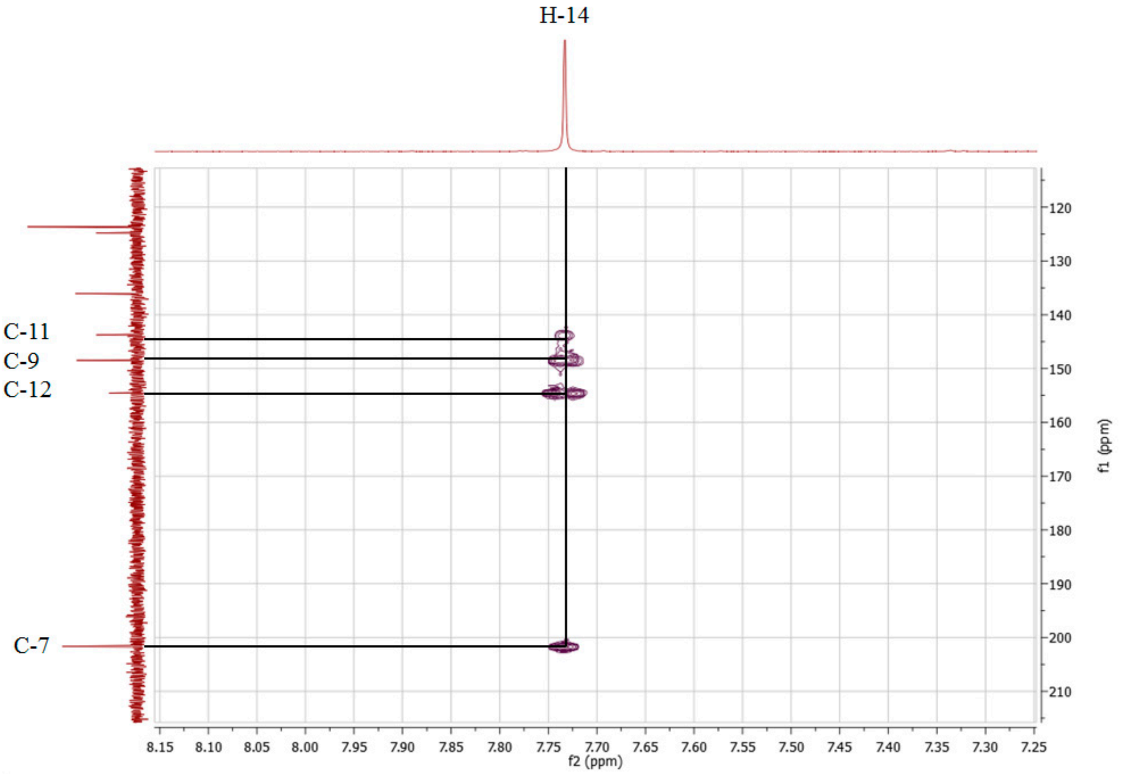

Figure S6. HMBC correlation from H-14 to C-7, C-9, C-11 and C-12 of compound **1** in methanol–*d*4 at 600 MHz.

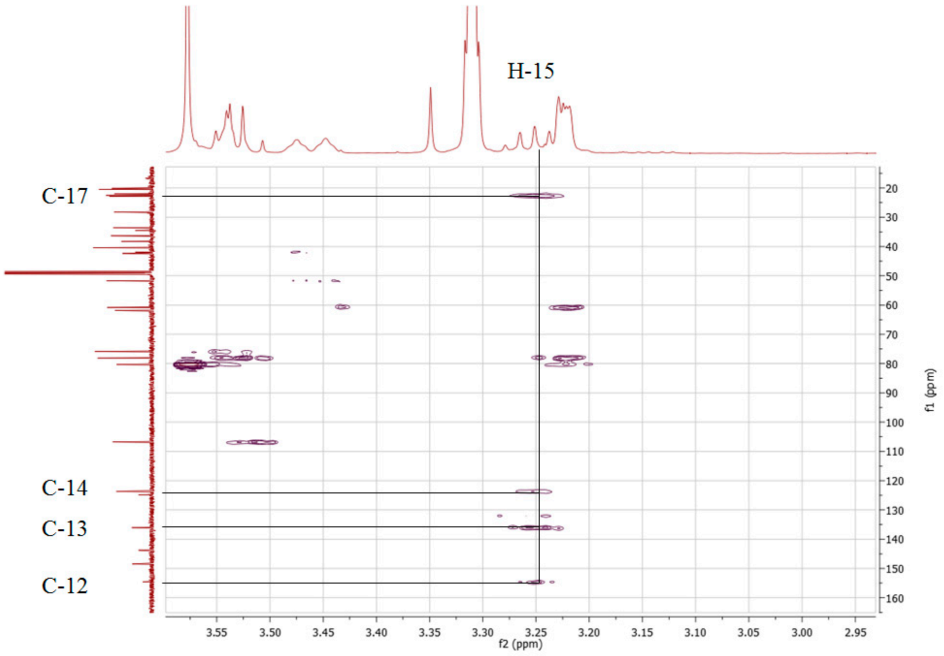

Figure S7. HMBC correlation from H-15 to C-12, C-13, C-14 and C-17 of compound **1** in methanol–d_4 at 600 MHz.

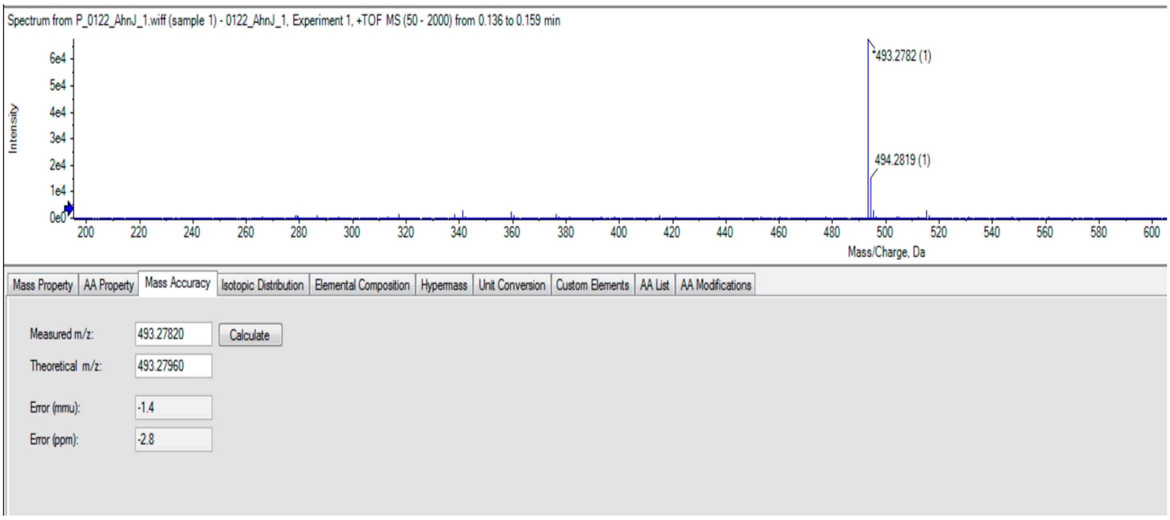

Figure S8. HRESIMS of compound **1** in positive mode.
